# Integrated knowledge translation in population health intervention research: a case study of implementation and outcomes from a school-based project

**DOI:** 10.1186/s12961-018-0351-8

**Published:** 2018-08-02

**Authors:** Jessie-Lee D. McIsaac, Tarra L. Penney, Kate E. Storey, Lori Sigfridson, Jane Cunningham, Stefan Kuhle, Sara F. L. Kirk

**Affiliations:** 1 Healthy Populations Institute, Dalhousie University, PO BOX 15000, Halifax, NS B3H 4R2 Canada; 2 Faculty of Education and Department of Child and Youth Study, Mount Saint Vincent University, 166 Bedford Highway, Halifax, NS B3M 2J6 Canada; 3 UKCRC Centre for Diet and Activity Research (CEDAR), MRC Epidemiology Unit, University of Cambridge School of Clinical Medicine, Box 111 Cambridge Biomedical Campus, Cambridge, CB2 0SP United Kingdom; 4 School of Public Health, University of Alberta, 3-300 Edmonton Clinic Health Academy, 11405 – 87 Ave, Edmonton, AB T6G 1C9 Canada; 5 Tri-County Regional Centre for Education, 79 Water Street, Yarmouth, NS B5A 1L4 Canada; 6 Western Zone, Nova Scotia Health Authority, 60 Vancouver Street, Yarmouth, NS B5A 2P5 Canada; 7 Department of Pediatrics, Dalhousie University, PO BOX 15000, Halifax, NS B3H 4R2 Canada; 8 Department of Obstetrics & Gynaecology, Dalhousie University, PO BOX 15000, Halifax, NS B3H 4R2 Canada

**Keywords:** Integrated knowledge translation, Research partnership, Population health intervention, Evaluation, School health, Well-being, Children

## Abstract

**Background:**

Integrated knowledge translation (IKT) is encouraged in population health intervention research (PHIR) to ensure the co-production of policy-relevant research, yet there is little published literature that reports its implementation and outcomes. The purpose of this study was to describe and evaluate the IKT approach used in a school-based PHIR project to understand how the research informed policy and practice and identify what influenced the IKT process.

**Methods:**

A case study approach was used to provide an in-depth description of the IKT process and understand the co-production and application of research evidence. Data were collected through document review, a survey with all elementary school principals in the school board (*n* = 18) following dissemination of School Reports and interviews with the IKT research team (including two researchers and three knowledge users).

**Results:**

Approximately half of the principals reported reading their School Report (52%) and almost all of these principals attributed the partial or full adoption, or implementation, of a new practice as a result of using the information (89%). Key themes related to the IKT process emerged across the interviews, including supportive relationships, role clarity, competing priorities and the complexities of population health interventions.

**Conclusions:**

The findings suggest that, while IKT can support policy and practice, it can be challenging to maintain engagement due to differing priorities and role ambiguity. Additional recognition, investment and research would enable better implementation of the approach, thereby bridging the gap between research, policy and practice.

## Background

Population health intervention research (PHIR) aims to contribute relevant, credible and timely evidence for decision-makers to improve policies and programmes that reduce the burden of illness at the population level [1]. Population health interventions are policies or programmes that are not led by researchers, often designed and implemented outside of the health sector, with the potential to shift the distribution of disease risk by addressing the underlying social, economic and environmental conditions in which people live [1, 2]. Although randomised controlled trials are commonplace in clinical settings, proponents of PHIR suggest that randomisation may not always be possible or ethical in certain real-world settings [3, 4]. Pragmatic trials, quasi-experimental designs or observational studies may provide better ways to understand under what circumstances an intervention might work to inform decision-making [3].

Although there has been increased investment in PHIR over the last decade, there are challenges that hinder the application of evidence relevant to policy and practice decisions to improve risk of disease at the population level (e.g. action often precedes research, outcomes take a long time to achieve) [5–7]. The Canadian Institutes of Health Research (CIHR) defines knowledge translation as “*a dynamic and iterative process that includes synthesis, dissemination, exchange and ethically-sound application of knowledge to improve the health of Canadians*” [8]*.* Integrated knowledge translation (IKT) is an approach suggested by the CIHR focusing on engagement of knowledge users (KUs) throughout the research process to co-produce research directly relevant to policy and practice change [8]. KUs can include stakeholders involved with policy and practice as well as the end recipients, such as families, that are influenced by the intervention. If successfully implemented, the IKT approach is expected to improve the likelihood that the research evidence produced will be used in policy and practice decisions [9]. IKT is often a requirement of research funding agencies like CIHR and studies are beginning to describe its implementation [10–14]. However, research is limited and few studies have evaluated the IKT approach to assess if the process has helped the achievement of expected outcomes [15]. The purpose of this study was to describe and evaluate an IKT approach used in a school-based PHIR project.

Although a traditional scientific paradigm may perceive the ‘knowledge-to-action gap’ [16] as the result of inadequate transfer or dissemination to KUs, there is increasing evidence that the application of research evidence may be less about how it is shared and more about how it is produced [9, 16]. This perspective suggests that research is not addressing the priority issues of KUs and that greater collaborative inquiry is needed between academics and KUs to leverage diverse perspectives and generate actionable evidence [9]. IKT offers an approach that focuses on the dynamic and collaborative exchange of information that crosses disciplinary boundaries, while functioning through two-way interactions between researchers and KUs to produce research relevant to the specific KU context [8, 17]. The assumption is that an emphasis on IKT will bridge the ‘knowledge-to-action gap’ by fostering frequent interactions between researchers and KU and building partnerships to ensure there is a clear understanding of the needs and context in which the research is conducted [9].

Collaboration and partnership between researchers and KUs is required for well-implemented IKT to ensure both parties are actively engaged in producing and applying knowledge [8, 17, 18]. Such partnerships can help to bridge the gap between research and practice to generate research that will help address complex population health problems [10]. Critical features for IKT include early involvement of all relevant stakeholders (researchers, practitioners, decision-makers), fostering open communication and realistic allowances for time, and aligning dissemination strategies with professional activities, educational resources and local expertise [10, 19, 20]. Thoughtful preparation and ongoing planning and problem-solving are also needed to sustain collaboration between researchers and KUs, considering the many obstacles that will inevitably arise (e.g. professional differences, competing agendas, divergent perceptions, and issues of power, trust and communication) [10, 21, 22]. Through working collaboratively, IKT partnerships support the development of research questions and data collection methods, analysis of data, interpretation and contextualisation of findings for policy and practice, and dissemination of results [9]. However, there is little research that has studied how the collaborative processes of IKT are implemented or might influence application of research evidence to inform policy and practice [11, 15, 23, 24].

### Research context

The small east coast province of Nova Scotia (Canada) offers a notable case for population health intervention research with a provincial health-promoting schools (HPS) initiative that has catalysed policy and practice change across school districts in the province. HPS is recognised globally as an effective multifaceted approach that involves an integrated curriculum, a supportive environment and healthy school policies, and is implemented with support from the whole school community [25]. Many school jurisdictions in Nova Scotia have adopted health promotion policies and guidelines as part of a broader comprehensive strategy to support healthier behaviours [26]. Building on existing relationships through earlier research collaborations, researchers met with partners in one school board to explore potential joint research ideas. Together, the objectives for the School Health and Well-being Project were developed to explore HPS implementation and its impact on school culture, student health and wellbeing, and its costs. This population-based study included students in grades 4–6 (9–12 years old) and their parents, across 18 schools in one rural school board with approximately half of schools having self-selected to implement a HPS approach (10 of 18). This school board includes a total of 23 schools (18 elementary) and encompasses three counties, representing a total population of 55,000 people (about 6% of the population of Nova Scotia), all of whom have a lower median income than the rest of the province. Data collection and results for the research has been published elsewhere; briefly, students and parents/guardians completed surveys on diet, physical activity and well-being, and an environmental assessment was conducted through a school audit and surveys with the principal and teachers [27].

### Purpose

The purpose of this study was to describe and evaluate the IKT approach used in the School Health and Well-Being project to support the co-production and application of research evidence. As well as understanding how the research was applied to inform policy and practice, this research also sought to identify what influenced the IKT process. It was expected that the findings from this research will help to inform future collaborative research opportunities for the local team and provide insight to understand how IKT should be supported to facilitate other PHIR projects.

## Methods

A case study approach was used to provide an in-depth description of the IKT process and evaluate the co-production and application of research evidence. Consistent with case study research, multiple sources of data were collected, including document review, interviews with the IKT research team (including researchers and KUs) and a survey with school principals [28].

### Sample and data collection

#### Document review

Minutes and notes from meetings and other interactions (e.g. presentations/workshops) between the researchers and KUs (*n* = 34) were reviewed to describe the interactions among the team throughout the project. Description of these meetings focused on who was involved, what was discussed, and where and when it took place.

#### Principal survey

Principals from all participating schools (*n* = 18) were invited to complete a short online survey about the use and usefulness of evidence produced from the research 1 year after receiving their individual school report. The survey focused on identifying conceptual (change in thinking) and instrumental knowledge use (change in policy/practice) adapted from a previously developed tool [29]. For example, participants were asked about their awareness, reception and thoughts related to the research evidence as well as its use for the adoption and implementation of school practices.

#### Interviews

A purposive sampling strategy [30] was used to collect information through interviews with research team members that used a guide developed to evaluate key dimensions of IKT partnerships (e.g. engagement in research process, communication, rapport, negotiation, commitment) [10]. A conversational style was used during the interviews to enable clarification and refinement of participant perspectives [31]. Key KUs representing the local HPS operation team from the school board and health authority and the principal researchers were invited to take part approximately 1 year following the delivery of the final report (*n* = 6). These individuals were the most engaged members of the research process and therefore the most likely to be able to provide a rich description of the IKT process. The interviews were conducted by the primary author, who was responsible for liaisons among the research team. It was important for this individual to conduct the interviews to ensure relevant probing and to maintain the integrity of relationships with KUs for future research projects.

### Data analysis

Documents from meetings were summarised in a spreadsheet to describe how IKT was implemented through meetings and interactions between researchers and KUs. Summary statistics were calculated for the survey questions to determine the percentage of schools reporting usefulness and use of the research evidence. With permission from participants, interviews were audio-recorded and transcribed verbatim. All identifying information was removed prior to analysis. The primary author used open coding strategies to identify codes and definitions were created to enable constant comparison when coding subsequent transcripts [32, 33]. Emerging themes and results were reviewed by all authors (which includes researchers and KUs) to ensure the results included a balanced representation of perspectives.

## Results

### Document review: description of the IKT approach

**Fig. 1:**
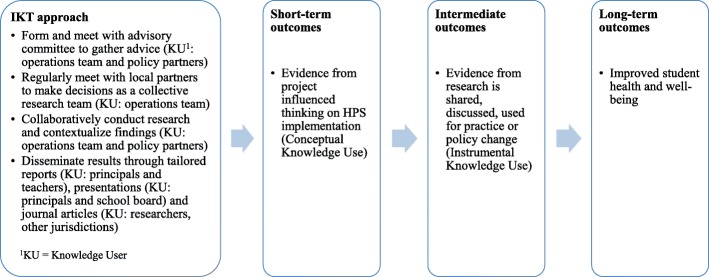
Logic model for integrated knowledge translation

IKT was used throughout this project to ensure the research was co-produced to help inform policy and practice. Figure 1 provides an overview of a logic model that describes how the hypothesised IKT approach would support the co-production and application of research evidence to inform policy and practice. It was assumed that short-term outcomes of the IKT approach would lead to conceptual knowledge use or changes in thinking and that immediate outcomes would be observed regarding instrumental use where evidence was used to inform policy or practice [34, 35].

A research advisory committee was established at the beginning of the research to provide guidance to the development of the research methods, instrument design, data collection and dissemination strategies. Terms of reference were developed to guide the mandate of the committee. The advisory committee comprised the lead researchers, practice partners from the local operations team for school health promotion and policy partners from the provincial government (including representation from both departments responsible for health and education). All advisory committee members were asked to provide advice and recommendations and act as key liaisons within their representative organisations. Membership was flexible so that additional committee members from the operations team and policy partners could attend depending on the issues being discussed. In total, the advisory committee met a total of six times between August 2013 and November 2014 with high participation by all partners (ranging from four to six policy/practice partners per meeting). Due to the rural location of the health promotion project studied, most advisory committee meetings were conducted via teleconference for members of the local operation team due to budget and time constraints.

The researchers also engaged local school principals and the elected school board prior to data collection to gather information on issues of importance to guide the research methods. To foster relationships and identify priority research actions, additional meetings were held with the researchers and local operation team members to make further decisions on the research process (34 in total). While the majority of these meetings took place by teleconference, the researchers travelled to visit with local partners as often as possible (seven local visits). Local evaluation assistants were trained to complete the data collection based on recommendations from the advisory committee. Findings were contextualised with KUs, and the results were shared through a variety of dissemination strategies with schools and the school board, including a confidential School Report with school-specific results on healthy eating, physical activity, mental well-being, and family engagement and presentations/workshops with school champions from HPS committees. A similar School Board Report was also co-developed with KUs for elected school board officials and a final presentation was delivered by the researchers. The results of the research have also been presented at a variety of academic and practitioner-based conferences, including one that was co-presented by researchers and KUs. Finally, corresponding with the publication of each peer-reviewed publication, a summary infographic is being disseminated to KUs to highlight key messages in an engaging format.

### Principal survey: use of research evidence by schools

All 18 school principals completed the online survey (100% response rate) on the use of results that were disseminated in the form of a School Report. A total of 83% of school principals were aware of their School Report (15 of 18) and 52% had either partially or fully read it (11 of 18). A range of uses was reported by those that read the report (Table 1). In terms of conceptual knowledge use, most principals that read their report felt research evidence in the School Report might be useful (91%) and was presented in a way they could understand (100%). Many of the principals that read their School Report also thought about the results and discussed it with others at their school (70%); however, fewer discussed it with other schools (30%). Many principals that read their School Report felt that it offered a new idea or way of thinking for how their support could encourage health promotion (67%). For instrumental knowledge use, almost all principals that read their School Report attributed the partial of full adoption of a new practice or the implementation of a health promotion practice as a result of their report (89%). For example, because of the identification of issues of student loneliness in their report, one school created the ‘Buddy Bench’ and introduced classroom lessons about empathy and friendships. Several schools had identified lower parental and community engagement and were exploring various ways to enhance engagement such as encouraging parents to run with their children or hosting cultural activities at the school.

**Table 1:** Outcomes of the integrated knowledge translation process as reported by school principals

	% (n)
Use of research evidence	
Aware of School Report (*n* = 18)	83% (15)
Partially or fully read School Report (*n* = 18)	52% (11)
Thought research evidence in School Report might be useful (*n* = 11)	91% (10)
Research evidence presented in a way they could understand (*n* = 10)	100% (10)
Conceptual knowledge use	
‘Sometimes’ or ‘often’ thought about the School Report (*n* = 10)	70% (7)
Made other school staff and partners aware of the School Report (*n* = 10)	70% (7)
Discussed the School Report with school staff and partners from your school (*n* = 10)	70% (7)
Discussed the School Report with school staff and partners from other schools (*n* = 10)	30% (3)
Cited the School Report in school documents (e.g. parent newsletters, continuous school improvement plans) (*n* = 9)	44% (4)
The School Report introduced a new idea or way of thinking to support health promotion at the school (*n* = 9)	67% (6)
The School Report changed beliefs about a particular approach to support health promotion (*n* = 9)	33% (3)
Instrumental knowledge use	
Attributed the partial of full adoption of a new practice or the implementation of a health promotion practice as a result of the School Report (*n* = 9)	89% (8)

### Interviews with lead research team members: use of research evidence by school board and influences on the IKT process

A total of five lead research team members (three KUs and the two principal researchers) agreed to take part in an interview, ranging from 30 to 55 minutes (only one KU did not respond to the interview request). Various positive impacts of the research were discussed by all participating research team members in terms of its support for policy and practice as well as building new evidence to inform HPS initiatives. One KU commented on the specific impact of the research on policy and practice: “*…* [it has] *given us a little more leverage with the board… an opportunity to engage administrators, more schools have come on board since that, since the research…*”*.* This KU also provided context on the use of research evidence by schools, which is complementary to the results from the principal survey results from the knowledge use survey, reinforcing that schools were using their school-level data: “*So every school has taken it, reviewed it, shared it with their school teams, have chosen priority areas out of it and then have created strategic action plans*”. The researchers also spoke about the impacts of the research through its contribution to the academic literature on HPS and population health interventions. Across all interviews, four key themes emerged related to the IKT process, including supportive relationships, role clarity, competing priorities and the complexities of population health interventions. Table 2 provides an overview of the themes and corresponding illustrative quotes.

**Table 2:** Themes emerging from interviews with research team

Theme	Illustrative Quotes
Supportive relationships	KU: “*I think what that has done is that has allowed us to be a little more candid with each other and, and have a level of honesty... I could pick up the phone and say I don’t quite understand this, walk me through it and feel completely comfortable… and the trust that, that we had built based on previous working relationships.*”
	KU*:* “*… if we did bring something up or we did, you know, kind of ask a lot of questions that you were willing to, you were willing to kind of go there.*”
Competing priorities	Researcher: “*I think* [delayed response from KU] *shows that you know, their priorities were very, very different from ours.*”
	KU: “*…the reality of the pressures that you have here* [in schools]*… you know it wasn’t about throwing up road blocks although it probably felt that way…*”
	KU: “…*we just weren’t able to really connect and get things finalized quickly, so whether it’s around the report and just kind of miscommunication around what we wanted in the report and what the report looks like…*”
	Researcher: “*…if we want to do this right then we need so many things, resources, you know, buy in, all those things… we need to be absolutely crystal clear what we’re doing in this, what the value is for everybody.*”
Role clarity	KU: “*… that was helpful… getting that constant kind, update of where things are going and what’s happening now.*”
	KU: “*I feel a little bit of guilt having been this kind of side line support area in some ways… I don’t know I just kind of I had a hard time during the entire process to keep in the loop.*”
	KU: “*…* [it would help to have] *an understanding of okay what are your time frames, what are your parameters as a researcher, um, you know and so that people understand where you fit into this space.*”
	Researcher: “*…perhaps weren’t clear about what we, what our role was and maybe again it’s probably two-way… we have to do a memoranda of understanding and everybody needs to have a clear identification of their roles…it’s like setting that front, but also reminding people along the way…*”
Complexities of the population health intervention	Researcher: “*But that to me was the actual what was so interesting about the work was that we were looking at this in a real-world environment… we’ve taken something that was happening anyway and we’ve looked at it and put it under a microscope and we’ve tried to sort of understand it and what we’re seeing is messy, its complex…*”
	KU: “*… when you have programs that you’re trying to establish as population based, when you’re in a system that doesn’t really recognize that way of working.*”

All research team members commented on the importance of supportive relationships between researchers and KUs throughout the research process. Participants commented that positive relationships were facilitated by prior experiences collaborating on research, personal relationships and the efforts by team members to communicate on a regular basis. Positive relations with the researchers seemed to allow more openness when challenges emerged and built trust in the research process. Both KU and researchers shared the perspective of the need for supportive relationships in the IKT process and identified that it was a priority to establish and maintain trust so KUs felt that they could openly inquire about the results. KUs commented that it was helpful that the researchers encouraged questioning to occur and were flexible to have discussions around particular areas of concern. One KU commented an appreciation that the whole team was learning and improving how they were working together as the project evolved.

Role clarity was discussed by both researchers and KUs. The researchers were identified as the leads for the project and were responsible for project implementation. The majority of meetings were organised by the researchers, who worked with KUs to engage them in the various phases of the research design and in the interpretation and dissemination of results. However, although there were terms of reference and organisational structures developed at the beginning of the project, all participants commented that there was sometimes lack of clarity of the roles. It was identified that this may have been worsened by the physical distance between the researchers and the KUs (~ 300 km) and that different KUs were more engaged in the data collection process due to their relationship with schools. Two KUs felt that greater clarity was needed on timeframes and parameters for different stakeholders involved, but one felt that the process was quite clear. The researchers talked about their responsibility in better clarifying roles but that it was important that KUs were reciprocally engaged in defining roles and had the capacity to truly engage and be accountable throughout the research process.

Although positive relationships were reported, it was evident that the researchers and KUs had competing priorities that influenced the IKT process. The researchers’ discussed this in relation to the frustration they experienced when they felt the research was not a priority for KUs as a result of tardiness or delayed response. Both researchers and KUs commented that the respective expectations, professional roles and priorities of each research team member may not have been fully understood. In particular, one KU commented on external stresses from a role working in the school board. Both researchers and KUs commented on the challenge of conducting research within school environments, especially engaging school stakeholders with competing academic priorities and obtaining consent from parents/guardians for student participation. Despite the best efforts by researchers and KUs, schools struggled with promoting uptake of participation in the research, which resulted in a lower response rate, negatively influencing the statistical power for the primary outcome evaluation. This was disappointing to both researchers and KUs. Researchers and KUs also commented on the challenges with co-producing dissemination products, which may have been influenced by the differing priorities. This was especially discussed by KUs in terms of the dissemination of the final School Board Report and presentation to the board. The researchers commented on the challenge they experienced in balancing the execution of the IKT approach with their obligation to produce scientific publications. Researchers noted that there needs to be greater value in the academic community and financial resources to support the IKT approach.

The complexity of the population health intervention that was studied was noted as a challenge for this project. Both researchers and KUs noted that HPS is an evolving initiative that needs to be adaptable to school circumstance. As a result, conducting research on the impact of the approach was perceived as difficult due to the variability in how it is implemented across different school contexts. The project tried to overcome the challenges in evaluating the complex population-level intervention with a multi-method design that included various data sources (surveys, school environmental scans, economic assessment). KUs commented on their appreciation for the diverse methods used, particularly the use of photos in the environmental scan, as it allowed for an illumination of differences that are more difficult to capture in traditional quantitative research. It is also important to note that KUs voiced interest in working with the researchers again, which suggests an overall positive relationship through this project.

## Discussion

This study sought to describe and evaluate the IKT approach used in a PHIR project by understanding how the evidence generated from research was applied to inform policy and practice and to identify what influenced the implementation of the IKT process. Overall, experiences from KUs and researchers were encouraging, and positive relationships were developed and fostered through the IKT approach. There were various positive impacts of the research evidence that was generated, including its usefulness for policy discussions and its application to inform practice changes in schools. Although only half of the schools had read their report, there was a similar number of schools participating in the HPS intervention [27]. Therefore, it could be assumed that those that read the report were already in the process of implementing changes as part of the HPS process. Those that did attributed the partial/full adoption, or implementation, of a new practice to using the information. Although this study did not ask schools to identify their HPS status, previous research has found that HPS schools were more likely to share and use research evidence [36]. Successes and challenges were noted by KUs and researchers in the implementation of IKT, including supportive relationships, role clarity, competing priorities and the complexities of population health interventions.

The findings of this study add to the findings from previous research on collaborations and partnerships in research [19, 20, 22, 23, 37] and provide a rich description of the process used to facilitate an IKT approach in school-based research. A research advisory committee was established to engage policy partners and the operations team early and throughout the research process and to foster open communication. The operations team also worked alongside the researchers to make key decisions related to the research process. These actions seemed to be helpful in strengthening relationships in this research, which builds on earlier studies that have reported that frequent and early interactions can help to ensure research is relevant and can bridge the gap by leading to increased use of research findings [10, 38, 39]. Kothari et al. [10] further discussed the need for preparation and ongoing planning to keep IKT partnerships moving beyond the obstacles that will inevitably arise. Further, they established indicators for partnerships to provide a transparent guide to develop and evaluate the success of a partnership from qualitative interviews with policy-makers and researchers involved with research partnerships, including those identified for ‘early’ or ‘mature’ partnerships and also specific indicators ‘common’ across research–policy partnerships in general [10]. Encouragingly, both researchers and KUs reported positive relationships, rapport, trust and commitment in this study, which is an indicator for a ‘mature’ partnership. Further ‘common’ indicators were evident, such as actions designed to enhance the collaborative research process and dissemination of findings (e.g. joint meetings, plans, practical formats and recommendations). However, ‘early’ indicators of partnership enhancement and negotiation of roles may have required more attention [10] as it was suggested that greater clarification of roles and timelines could have enhanced communication and engagement of KUs.

The complexities involved with PHIR were also noted in relation to IKT. HPS is a complex intervention implemented across heterogeneous and dynamic school contexts [40]. We have previously described the implementation of the programme in the school board, which began prior to the commencement of this study [27]. The concept of ‘action preceding science’ among population health interventions has been previously discussed in the literature in terms of the challenges of PHIR [7]. Although there is a benefit to embed research into existing interventions (such as HPS in the school board) to generate meaningful information on potential improvements, it can be difficult to assess HPS interventions due to their variable nature, especially when any impact on behavioural or health-related outcomes can take a long time to detect [7]. A further challenge of implementing PHIR in schools was also noted in the disappointing response rate from parents. However, this has been noted to be a challenge in school-based research considering the increasing demands on school curriculum time, especially when active consent is required (i.e. parents having to return a signed form) [41]. Future research could spend more time promoting the research with teachers and parents to encourage better response or consider passive consent processes. These challenges of evaluating natural experiments (as result of action often preceding the science) can be difficult to emphasise among KUs or decision-makers that may have different expectations and goals for engaging in research [7, 10, 21, 42]. Although this obstacle is important to note, researchers and KUs may also use the collaboration for different purposes and variations in goals may not be problematic [11]. Researchers in this study commented on the effort required to manage priorities among KUs. Although improved communication about roles, timelines and professional priorities may have mitigated obstacles and limited diverging expectations, researchers perceived this to be difficult with limited resources and scholarly recognition for IKT. For example, the time-limited and project-specific nature of funding can sometimes be difficult when trying to build more sustainable partnerships, particularly in PHIR, where it may take longer for outcomes to emerge [7]. Further, although valuing of IKT may be different across institutions, the need for an academic culture that supports ‘engaged scholarship’ (advancing both theory and practice through authentic collaboration) has previously been discussed as needing to be integrated into tenure and promotion guidelines and faculty reporting [43–45]. More systematic research on these academic challenges is warranted to provide guidance on the support that is needed to facilitate IKT.

### Limitations

This case study provides a rich description of an IKT approach used in a population-level research project in schools. Although the study results are not meant to be generalisable, there are limitations that may influence the transferability to other settings. First, there may have been selection bias and social desirability bias in the self-report survey that assessed knowledge use in schools. Further, although schools reported usage of their reports to inform practice changes, the extent to which the information influenced these practice changes (compared to other information) is not known. However, through triangulation of data collected as part of the interviews and through further discussion with KUs, it was acknowledged that the school reports were being used by school principals to inform changes. Second, not all KUs and researchers involved with the project were interviewed as part of the study, with several KUs no longer in the same position as a result of recent reorganisations of the health and education systems in the province. Although not all experiences were fully explored, those involved were identified as the primary researchers and KUs involved with the project. There may also be potential bias through self-evaluation, with some of the authors also taking part as participants in the interviews. A balanced perspective from both researchers and KUs was assured by the comprehensive authorship team, which includes representation from both. Finally, although it was important for the primary author to lead the interviews to support ongoing partnership development for research projects, it is possible that participants may not have felt comfortable to disclose negative experiences with the study. To address this limitation, participants were assured that all perspectives were valued and all experiences (positive and negative) would support improvements in the research process. Two KUs are also authors on this paper and have verified the interpretation of results presented.

## Conclusions

Although an IKT approach is commonly required and advocated for in population health research, there is little published research that describes its implementation and the resulting impacts on policy and practice. This study provides a description of the process and outcomes of IKT in a school-based research project through a case study that describes the actions influencing partnership development and application of the research evidence that was generated. This study suggests that ongoing exchange between researchers and KUs is important to ensure integration of relevant results to inform practice changes in schools, policy development for the school board and transferable knowledge for other jurisdictions. However, although positive outcomes were reported, the findings of this study also suggest that IKT can be challenging, and that dedicated resources are needed to maintain engagement and negotiate roles with KUs that have different priorities and expectations from researchers. Additional recognition of the importance of IKT in the academic community and subsequent development of resources would enable better implementation of the approach. Further research is also needed to identify key actions that support IKT, thereby facilitating the potential for impact on policy and practice.

## Abbreviations

CIHR: Canadian Institutes of Health Research
HPS: health-promoting schools
IKT: integrated knowledge translation
KU: knowledge user
PHIR: population health intervention research
